# Individual and combined impacts of water immersion, steaming at 100 °C and hot air drying on the cashew nut shelling process

**DOI:** 10.1007/s13197-025-06344-6

**Published:** 2025-06-05

**Authors:** Mamadou Tanou Diallo, Martial Madoumier, Joseph Bassama, Léa Ollier, Arnaud Chapuis, Antoine Collignan

**Affiliations:** 1 Qualisud, Univ Montpellier, Avignon Univ, CIRAD, Institut Agro, IRD, Université de La Réunion, 1101 av. Agropolis, cedex 5, Montpellier, 34093 CS 24501 France; 2 CIRAD, UMR Qualisud, Montpellier, F-34398 France; 3 Université Gaston Berger, Saint-Louis, Sénégal

**Keywords:** Cashew nuts, Shelling, Weakening, Moisture content, Artificial drying, Whole kernel yield

## Abstract

Shelling cashew nuts is a highly labour-intensive process that often results in broken kernels, reducing efficiency and economic value. Improving shelling techniques is crucial for increasing whole kernel yield, reducing waste, and enhancing sustainability in the cashew industry. In this study, the aim was to investigate the impact of pre-treatments (water immersion, steaming and artificial drying) applied alone or in combination to weaken the nut and facilitate shelling. An experimental approach incorporating microtomography was used to identify the importance of each step and the factors controlling the process. The results show that water content is the main factor influencing whole kernel yield, shelling rate and shell toughness. Hydration during soaking makes the shell more flexible and reduces kernel cracking, while steaming intensifies this effect. Rapid drying keeps the kernel hydrated and makes the shell brittle, improving the production rate. The study suggests that steaming alone might sufficiently saturate the shell, eliminating the need for soaking. In addition, artificial drying reduces the duration of the process. The results obtained provided a better understanding of the process control variables with a view to its improvement. Further studies on process modelling could provide insights into optimising the yield of whole kernels.

## Highlights

- A study of the cashew nut shelling process is carried out;
- Pre-treatment has a decisive impact on shelling quality;
- Microtomography imaging reveals structural changes in cashew shells during pre-treatment;
- Lignocellulosic and starch transformations improve shell brittleness and kernel resistance to stress;
- The mechanisms of cashew nut embrittlement have been elucidated.

## Introduction

Cashew nuts, which are the third most produced nut in the world (INC, 2020), are grown mainly in West Africa, which in 2018 rose to become the world’s leading producer, with 1,795,000 tonnes of raw cashew nuts being marketed, representing 49% of global supply (Ricau 2019). Despite this high production, the quantity of raw cashew nuts processed locally barely reaches 10% of production (Ricau 2019; INC 2020; Kolliesuah et al. 2020). Also, given that the cashew nut market will continue to grow for several years to come, in particular, due to cashew kernels being considered as “healthy” (Mandal 2019; Mah et al. 2017), several West African countries are making efforts to promote local processing of raw cashew nuts (Kolliesuah et al. 2020). This implies a better understanding of the various stages involved in processing this product before being able to design a suitable process and produce quality kernels.

**Fig. 1:**
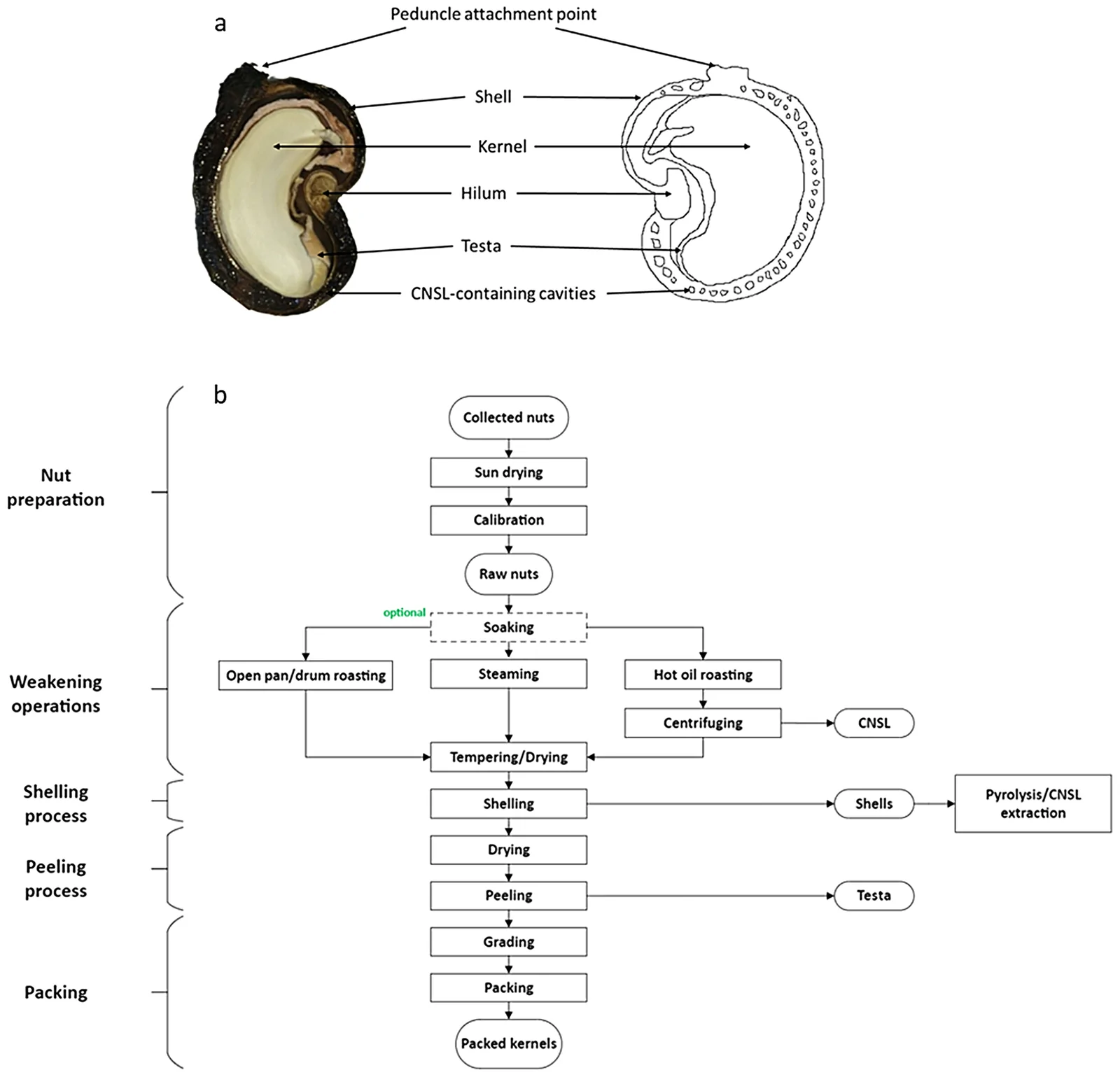
**a** - Photo and schematic drawing of a cashew nut in longitudinal section, **b** - Cashew nut transformation processes

The cashew nut is defined as a kidney-shaped, ash-green achene with a shell consisting of a spongy epicarp, a hard endocarp and a softer mesocarp containing the dark, caustic, viscous cashew nutshell liquid (CNSL). Inside the shell is the kernel, also kidney-shaped, covered on the outside with a thin reddish-brown skin, called the testa, which protects and separates it from the endocarp. In terms of mass proportion, the shell typically represents about 70% of the total nut mass, with 35–40% being constituted by CNSL; the kernel and testa together occupy the remaining 30% of the total nut mass on average (Azam-Ali and Judge 2001; Balasubramanian 2001; Lautié et al. 2001; Ogunsina and Bamgboye 2014; Muniz et al. 2013). Figure 1a shows the internal structure of a cashew nut, comprising the kernel, testa and shell. The cashew kernel constitutes the edible part of the nut, which is widely appreciated as a snack, whether eaten raw, roasted or salted. It is also used as an ingredient in a variety of baked goods, confectionery and savoury dishes.

The process of extracting cashew kernels from raw cashew nuts remains laborious and time-consuming. It consists of a series of operations (Fig. 1b), generally beginning with the weakening of the nut shells, followed by shelling, drying of the kernels, peeling, grading and packing.

Among these stages, shelling, which aims to separate the kernel from its shell, is the most delicate. This operation has to be carried out with great care in order to preserve the integrity of the kernel and avoid any contamination by the CNSL, thus justifying the need, prior to this, to weaken the shells of the raw nuts.

Weakening generally involves two stages: heat treatment followed by drying. Heat treatment softens the shell and promotes the detachment of the kernel from the inner surface of the shell. The drying that follows is generally carried out in the open air in the shade to harden the shells and make them brittle. This makes it easier to open the shells and separate the kernels during shelling, reducing the production of broken kernels while improving the shelling rate (Azam-Ali and Judge 2001; Lautié et al. 2001; Balasubramanian 2006; Ogunsina and Bamgboye 2014; Tran et al. 2020).

Several authors, including Azam-Ali and Judge (2001), Ogunsina and Bamgboye (2014) and Tran et al. (2020), have mentioned adjusting the moisture content of raw nuts by immersing them in water before the heat treatment. According to Azam-Ali and Judge (2001), this has the effect of increasing the water content of the kernel making it more flexible and therefore less likely to crack during shelling. However, this operation is rarely encountered in industry.

Although there are various types of heat treatment applicable to nuts, two methods are widely used in cashew nut processing plants. These are oil roasting - also known as the immersion method in a heated CNSL bath - and steaming. The cashew nut roasting and steaming processes are shown in Fig. 1b.

Oil roasting involves immersing the nuts in a heated bath of CNSL between 180 °C and 190 °C for approximately 2 min. An advantage of this method is that it facilitates the extraction of CNSL, thus allowing for economic valorisation of this liquid. This is not the case with the open-air roasting method, where the nuts are heated to the smoke point of the CNSL, resulting in significant losses of both CNSL and kernel quality due to browning (Azam-Ali and Judge 2001; Tkatchenko 1949). Furthermore, oil roasting, although not widely used in industrial settings, produces low-value kernels. It leads to kernels that are coloured or browned (white, pale or ivory kernels are preferred), and generates a high rate of broken kernels, which devalues them compared with whole kernels. In addition, the roasting method is polluting because of the thick, acrid fumes it generates, which are hazardous to the health of operators (Azam-Ali and Judge 2001; Ogunsina and Bamgboye 2014; Tran et al. 2020).

The steaming method represents an alternative to the roasting method. It is still widely used by cashew nut processing plants. The advantage is that it is less polluting (Balasubramanian 2006; Ogunsina and Bamgboye 2014; Tran et al. 2020). Several authors, including Balasubramanian (2006), Kosoko et al. (2009), Mohod et al. (2010) and Dhanushkodi et al. (2016), have suggested subjecting raw cashew nuts to steam pressures typically ranging from 4 to 7 bars for durations of 20 to 40 min. Tran et al. (2020) showed that steaming with steam at 100 °C (steam at atmospheric pressure) for 38 min constitutes a good compromise, allowing sufficient weakening of the nuts while preserving the quality of the kernels during shelling.

After heat treatment, the steamed nuts are dried directly in ambient air to harden and make the shell brittle. Drying is generally carried out in the open air in the shade for periods ranging from 12 to 24 h (Balasubramanian 2006; Mohod et al. 2010; Ogunsina and Bamgboye 2014; Dhanushkodi et al. 2016). Few studies (Tran et al. 2020; Kosoko et al. 2009) have looked at the drying of steamed nuts under controlled conditions.

Although some studies have already been carried out on the shelling process (Balasubramanian 2006; Ogunsina and Bamgboye 2014; Tran et al. 2020), the quality of shelling in terms of whole kernel yield with an optimised shelling rate still remains a major difficulty in small and medium-sized enterprises (SMEs) in West Africa, particularly in Senegal and Burkina Faso.

To our knowledge, no research has explored the potential effects of hydration by immersion in water, combined with steaming at 100 °C and controlled drying in a dryer, on the ease of the cashew nut shelling process. Therefore, the objective of this study is to understand the individual and combined effect of these unit operations on whole kernel yield, shelling rate and shell breaking strength. This study will also examine the impact of these operations on the morphology of the whole cashew nut, shell and kernel.

## Materials and methods

### Sample preparation

Raw cashew nuts, sourced from the Casamance region in Senegal, with lengths ranging from 22 to 30 mm, were used as the raw material for this study. These nuts size, most commonly found and processed in this region, are classified as small-sized according to the criteria established by Kilanko et al. (2020).

Their initial moisture content was 0.77 (dry basis).

### Experimental design

**Fig. 2:**
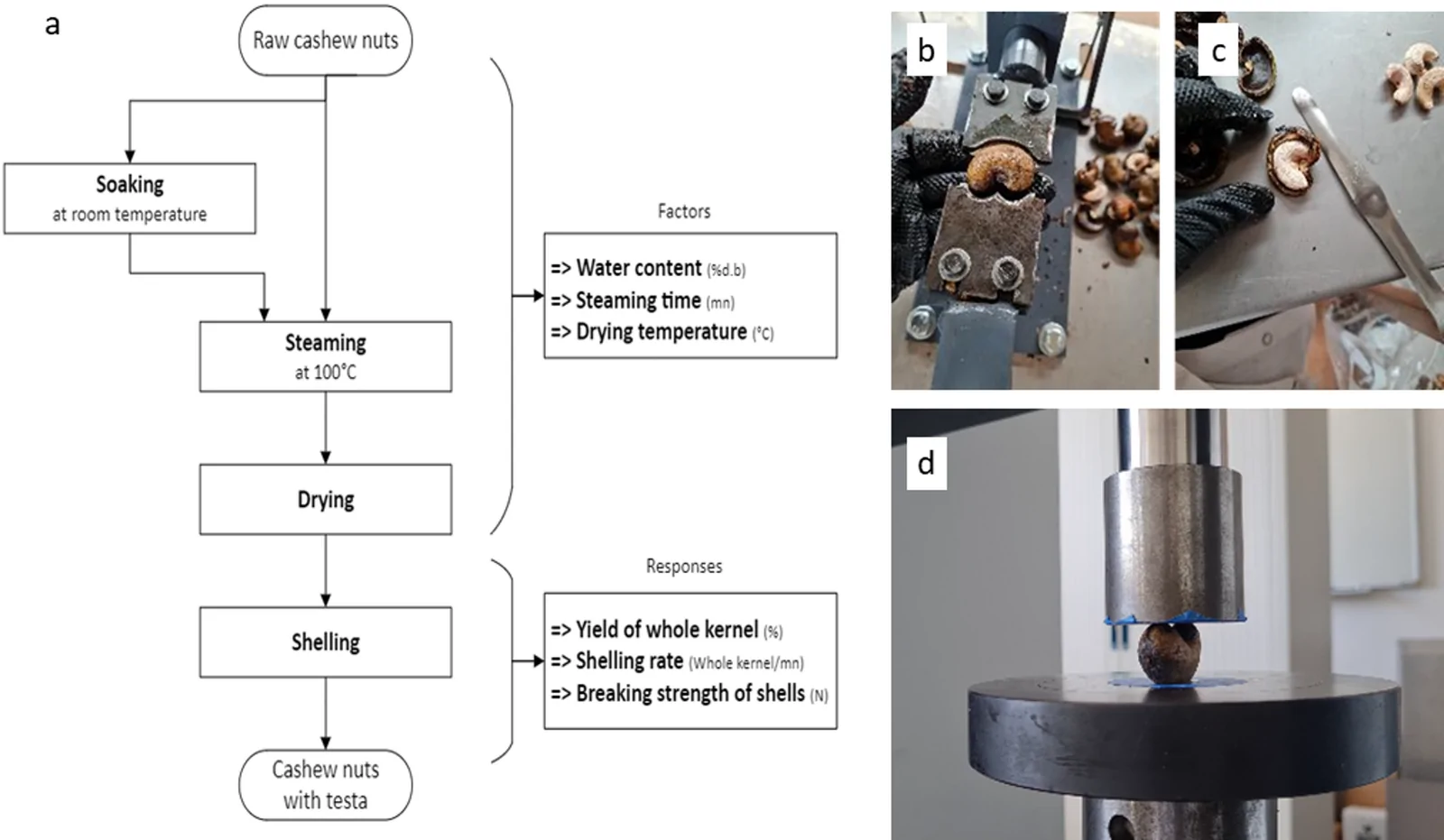
**a** - Weakening process studied with the factors of the factorial plan and the responses to be measured during shelling, **b** - Type of shelling performed in this study using a pedal-operated sheller (Pasen Machine YGT-1), **c** - Tool used to fully extract the kernel, **d** - Positioning of the cashew nut sample between the two parallel compression plates of the rheometer (Adamel Lhormagy - DY32, 10 kN) during the measurement of shell rupture force

A 2-level factorial design with 3 factors and a repeated central point 3 times was used to study the influence of soaking at room temperature, vapour steaming at 100 °C, and hot air drying on the shelling quality. Figure 2a illustrates the laboratory-scale process implemented to study the influence of these pretreatment operations, with a period of 5 to 10 min between each application of operation. The factors studied are the water content associated with soaking, the steaming time and the drying temperature. The measured responses are the whole kernel yield at shelling, the shelling rate of whole kernels, and the breaking strength of shells.

Table 1 presents the ranges of variation for the factors along with their upper and lower limits, determined from the literature. The water content was set at 20.0% db as the upper limit, according to the article by Ogunsina and Bamgboye (2014), while the initial water content of the nuts (7.7% db) constitutes the lower limit and corresponds to the typical water content of raw nuts. The duration of steam treatment at 100 °C was established between 30 and 60 min based on the studies by Kosoko et al. (2009), Ogunsina and Bamgboye (2014), and Tran et al. (2020). Regarding the drying temperature, the limits are set at 30 °C as the lower limit, after steaming, and 60 °C as the upper limit, to assess the potential benefits of increasing the temperature to reduce the drying time.

**Table 1:** Factors studied with their units, symbols, high and low levels for the factorial plan and literary references

			Levels		
Independent variables	Units	Symbols	-1	1	References
Water content	% db	X_1_	7.7	20.0	Kosoko et al. (2009); Ogunsina et Bamgboye (2014); Tran et al. (2020); Azam-Ali et Judge (2001) ;
Steaming time	mn	X_2_	30.0	60.0	
Drying temperature	°C	X_3_	30.0	60.0	

### Soaking procedure

To provide the required amount of water to achieve the desired water content according to the points of the experimental design, the nuts are soaked in water at room temperature (25 °C). Achieving the desired water content is determined using soaking kinetics established for this purpose. At the end of soaking, the nuts are wiped with absorbent paper to remove excess water from the surface.

### Steaming procedure

The nuts are steamed at 100 °C, and the steaming times depend on the instructions defined in the experimental design. Couscous steamers and induction plates (Electrolux, 2 kW) were used for the operation.

### Drying procedure

The steam-treated nuts are dried in a pilot convection air dryer (Union Technique Aquitaine brand) equipped with 20 trays having a total surface area of 2.6 m^2^, operating between 25 and 75 °C with an adjustable airspeed between 1 and 3 m/s. Drying is carried out according to the established drying temperatures in the experimental design, with an airspeed of approximately 3 m/s. The relative humidity of the air was measured but not controlled. It varied depending on the drying temperature: 12 ± 1% at 60 °C, 22 ± 3% at 45 °C, and 42 ± 3% at 30 °C.

### Shelling procedure

The nuts were shelled using a manually operated pedal sheller (Fig. 2b), the Pasen Machine YGT-1, which functions based on the compression-shear principle. The operator controls the force applied by pressing the pedal. The compressive force holds the nut in place, while the shear force cracks and opens the shell, allowing kernel extraction. Since the shelling process is manually controlled, precise force values are not measured. However, to ensure uniform force application, the same operator conducted all shelling trials. In cases where the kernel remains stuck (Fig. 2c), an additional manual finishing step using a small tool is required. To maintain consistency, 25 nuts were shelled per experimental point under uniform conditions.

#### Determination of whole kernel yield

The whole kernel yield Yield_wk_ is calculated as the ratio of the number of whole kernels 𝑁_wk_ obtained from the 25 shelled nuts.

1 $$\:\begin{array}{c}{Yield}_{wk}=\frac{{N}_{wk}}{25}\end{array}$$

#### Determination of the whole kernel rate

The whole kernel rate at shelling Rate_wk_ is calculated by dividing the number of whole kernels N_wk_ obtained at shelling by the time D required to obtain this number of whole kernels. Two steps are considered in this process: cracking the nuts by the sheller and separating the kernel from the shell using a small tool.

2 $$\:\begin{array}{c}{Rate}_{wk}=\frac{{N}_{wk}}{D}\end{array}$$

### Measurement of shell breaking force

The rupture force represents the force required to crack the nuts. It is measured using a rheometer (Adamel Lhormagy– DY32) with a capacity of 10 kN. This measurement is performed on 25 nuts at each point of the experimental design. The transverse position, as shown in Fig. 2d, was considered for the positioning of the nuts on the machine (Ogunsina 2013; Balasubramanian 2015). The probes consist of two metal plates. The descent speed of the upper probe was set at 2 mm/s. A deformation limit of 5% was set as the indicator of nut-cracking detection.

### Measurement of moisture content before and after each operation

Before and after each soaking, steaming and drying operation, the moisture content of the nuts, kernels and shells was measured using the oven drying method (BS ISO 11093-3:1994 1995).

### X-ray microtomography measurement of nuts

To comprehensively track the structural modifications undergone by the seed during various pre-treatment operations, three nuts were randomly selected from a batch of 25 nuts and individually scanned using X-ray microtomography in their raw state (a), after soaking (b), after steaming (c), and after drying (d).

Microtomography was conducted using a high-resolution scanner, SkyScan 1272 (Bruker©, Kontich, Belgium), with a nominal resolution of 25.4 μm employing an aluminium filter of 0.25 mm thickness. X-ray beam parameters included a voltage of 65 kV and an intensity of 153 µA, chosen to ensure a constant 30% signal transmission. The X-ray source power (*P* = U.I) was maintained constant at 10 W. A camera pixel binning of 1008 × 672 was applied. The scanning orbit was set at 180° with a rotation step of 0.9°. Bruker software tools were used for image processing.

The GPU-accelerated NRecon^®^ software was employed to reconstruct scan projections into images, applying Gaussian smoothing, ring artifact reduction, and beam hardening correction. 3D volume renderings were generated using the RGBA transfer function in CTVox^®^ software. Different slices, based on the selected angle, were generated via DataViewer^®^ software. Image analysis was conducted using CTAn^®^ software. 3D morphometric parameters such as volume were calculated for selected volumes of interest (VOIs), namely the whole nuts, shells, and kernels. Specific task lists were developed to perform VOI selections, binary segmentations, and morphometric analysis of the twelve samples.

### Statistical analysis

Statistica 8 software was used as the tool for statistical analysis of the experimental design. The effects of factors were considered significant at *p* < 0.1. The underlying linearity assumption of the chosen experimental design was validated by analysing the significance of curvature defined as the difference between the theoretical mean proposed by the model and the experimental mean. Model linearity is validated for *p* > 0.1.

## Results and discussion

### Results

#### Effects of various factors on shelling performance indicators

Before being shelled, the cashew nuts are weakened by the unit operations shown in the diagram in Fig. 2a with the experimental design processing conditions set out in Table 1. Table 2 presents the results of the statistical analysis of the experimental design. For the three responses, the R2 values are between 0.88 and 0.98, showing a good fit between the experimental results and the model.

**Table 2:** Analysis of variance of the results of the experimental study of the different measured responses of the factorial design (regression coefficients and statistical significance)

	Measured responses			
	Yield of whole kernels (%)	Shelling rate (whole kernels/mn)	Shell rupture force (*N*)	
R^2^	0.879	0.980	0.941	
Mean	0.800	2.376	861.625	
Curvatr.	0.013^−^	-0.028^−^	-33.292^−^	
Water content X_1_	0.090^**^	0.762^***^	-55.125^***^	
Steaming time X_2_	0.010^−^	0.231^*^	2.125^−^	
Drying temperature X_3_	0.050^−^	0.055^−^	10.625^−^	
X_1_ by X_2_	0.060^−^	0.345^**^	-5.625^−^	
X_1_ by X_3_	-0.020^−^	-0.104^−^	9.375^−^	
X_2_ by X_3_	-0.040^−^	-0.175^*^	0.125^−^	

***p < 0.01; **p < 0.05; *p < 0.1;^−^*p > 0.1*

##### Whole kernel yield

Statistical analysis (Table 2) shows that whole kernel yield is influenced exclusively by X_1_ moisture content, with a significant positive effect (*p* = 0.046). Furthermore, no interaction between the various factors studied had a significant effect on whole kernel yield. Given that the value of the non-linearity indicator (curvatr.– software term) is not significant, we can conclude that there is a linear correlation between whole kernel yield and X_1_ water content.

Whole kernel yield ranged from 64 to 96%, corresponding to moisture contents of 7.7% db and 20.0% db respectively, with an average of 80%. These yields are of the same order of magnitude as those in the study by Ogunsina and Bamgboye (2014), which range from 38 to 97% depending on size, moisture content, and time of exposure to steam at 7 bar. However, they are lower than those of Balasubramanian (2006), who examined the influence of steam exposure time at 7 bar and open air drying time, and Tran et al. (2020), who studied the steaming temperature, steam exposure time, vacuum drying temperature and time, and open air drying time of large nuts (length greater than 32 mm). In these two studies, the yields of whole kernels were between 90% and 96%.

##### Rate of whole kernels during shelling

The factors influencing whole kernel yield during shelling (Table 2) are moisture content X_1_ with a significant positive effect (*p* = 0.001) and drying time X_2_ with an equally positive effect (*p* = 0.052). The results also indicate the possibility of interaction effects between moisture content X_1_ and drying time X_2_, as well as between drying time X_2_ and drying temperature X_3_, with positive and negative effects respectively. Furthermore, the non-linearity hypothesis was not significant for this response.

##### Breaking strength of cashew nut shells

The water content X_1_ emerged as the only factor influencing the breaking strength of cashew nut shells. Its impact is negative with a *p* = 0.008. Furthermore, the interaction effects and the non-linearity hypothesis were not significant for this response (Table 2).

The breaking force shows a range of variation from 773 N to 933 N, with an average of 853 N. This average exceeds the values observed in research carried out by Ogunsina (2013) and Balasubramanian (2015) investigating shell toughness, carried out under similar conditions, with respective forces of around 309 N and 377 N. However, these values are within comparable orders of magnitude. The differences found between the breaking strengths reported in this study and those found in previous research could be attributed to several factors, including the variety of nuts used, the measurement device employed and the operating conditions. Ogunsina (2013) and Balasubramanian (2015) worked with descent velocities of 0.8 mm/s and 1 mm/s, respectively, while in this study a velocity of 2 mm/s was applied.

#### Evolution of the kernel and shell water content

**Fig. 3:**
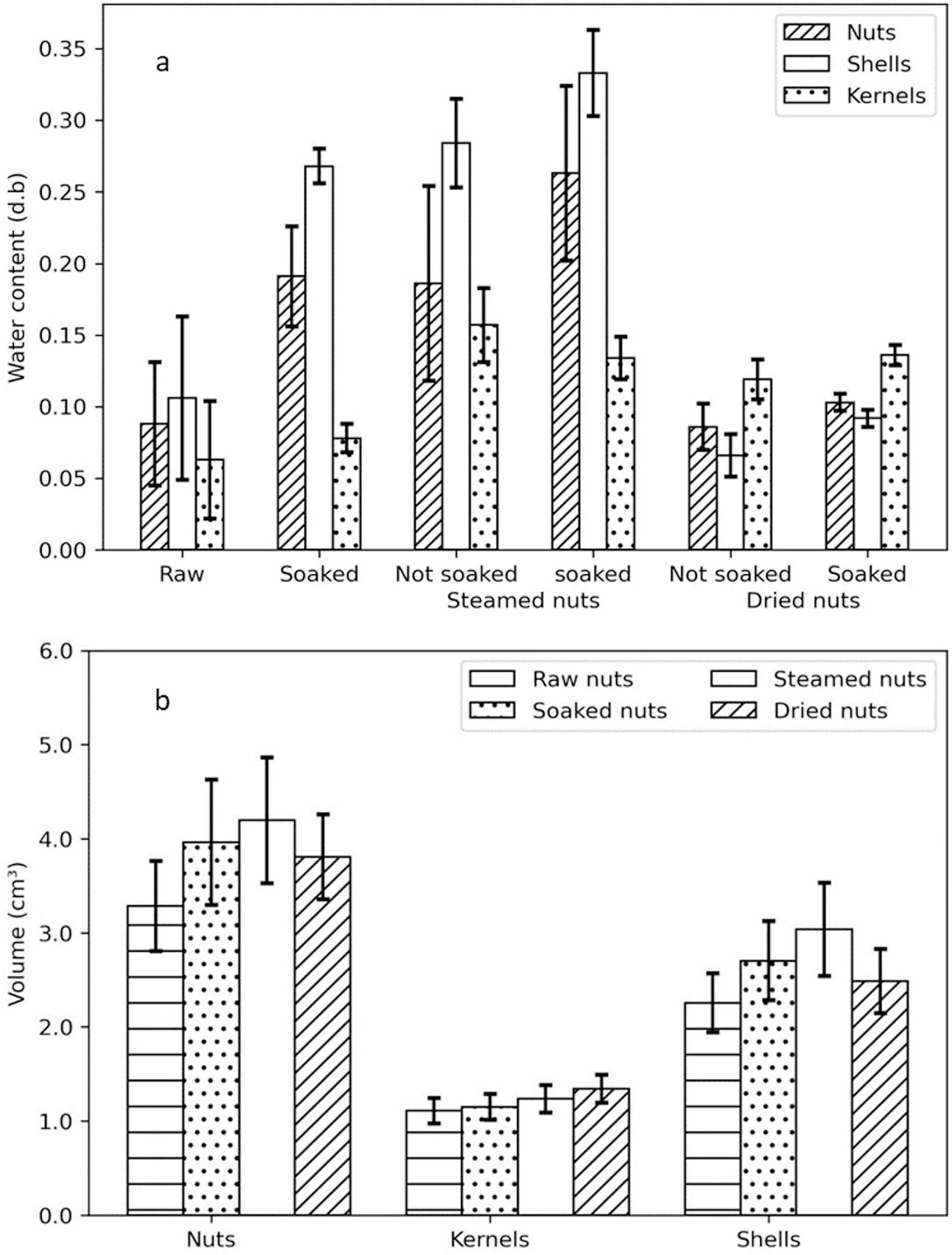
Evolution of water content (**a**) and volumes (**b**) of nut, shell, and kernel during soaking, steaming, and drying operations

Figure 3a shows the evolution of the water content, accompanied by the 90% confidence intervals, for the whole nut, the shell and the kernel for the raw nut and after each pre-treatment operation. Statistical analysis revealed that there was no significant difference between the kernel and the shell in the case of raw nuts. However, a significant distinction emerges between the kernel and the shell following the pre-treatment operations.

Overall, the water content of the whole nut increases during the successive soaking and steaming operations. This increase is explained by the significant hydration of the shell. However, the degree of hydration of the shell obtained after steaming is no greater when the nut is soaked beforehand. This would suggest that the fibrous structure of the shell would have become saturated with water during soaking and that further steaming would not provide any additional water. Figure 3a also shows that steaming alone is sufficient to saturate the shell with water.

The soaking process does not significantly increase the water content of the kernels. On the other hand, the subsequent steaming does significantly hydrate the kernel. This can be explained by the transfer of water between the shell (already saturated) and the kernel during steaming. On the other hand, simple steaming without prior soaking quickly saturates the shell and also allows water to be transferred from the shell to the kernel. It should be noted that the degree of hydration of the kernel is identical after steaming whether or not it has been pre-soaked.

During drying, the nut dehydrates significantly and returns to its initial moisture level. This dehydration is explained by a net drying of the shell, while the kernel retains a degree of hydration higher than its original level. Finally, no difference was observed between the different compartments of the nuts, whether soaked or not.

To sum up, from a hydric point of view, soaking or steaming operations allow rapid water impregnation of the shell. Secondly, it is during the steaming process that the kernel is hydrated. This hydration of the kernel persists during drying, when the shell regains its initial water content. In conclusion, adding a soaking operation contributes nothing in terms of hydration of the different parts of the cashew nut.

#### Results of microtomographic observation of the impact of pre-treatment operations

**Fig. 4:**
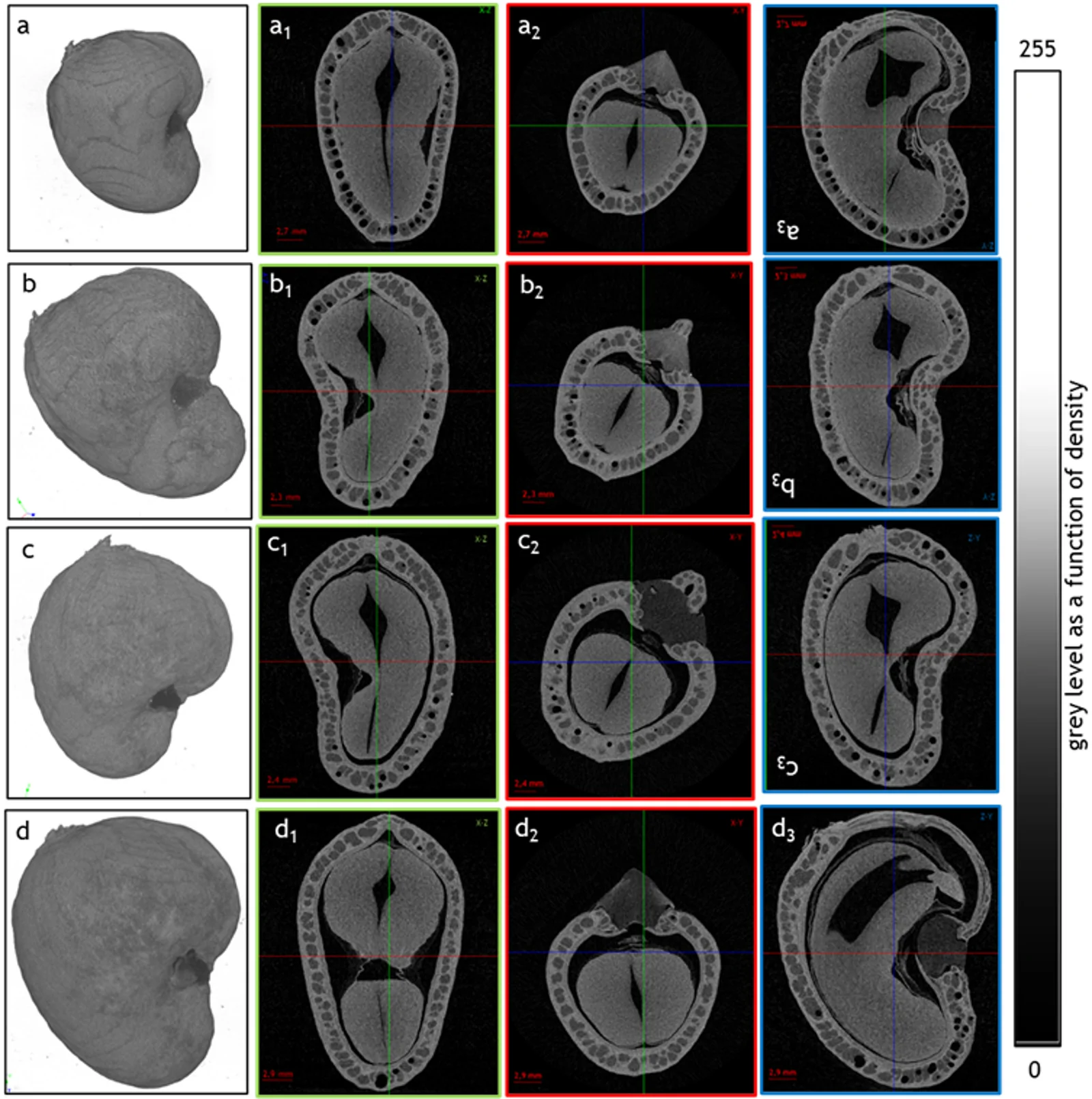
Cross-sections at different orientations of a cashew nut after each process: **a** - Raw nut; **b** - Nut after soaking; **c** - Nut after soaking and steaming; **d** - Nut after soaking, steaming then drying

Figure 4 shows cross-sections at different orientations of a cashew nut following the application of pre-treatment operations to ensure good weakening while maintaining the integrity of the kernels. These pre-treatments consist of soaking for 15 h at 27 °C, followed by steaming at 100 °C for 1 h, then drying at 60 °C with a relative humidity of 11% and a drying air speed of 2 m/s. Cross-sectional images of the raw nut (Fig. 4a_1_, a_2_ and a_3_) reveal strong adhesion between the kernel and the shell, with the exception of the hilum area, which is the concave area on the shell surface (Fig. 1a). In addition, the kernel has a space between its two cotyledons. Some parts of the shell also have empty cavities, that are not filled with CNSL. The cross-sectional images of the soaked nut (Fig. 4b_1_, b_2_ and b_3_) show no structural changes between the kernel and the shell compared with those of the raw nut (Fig. 4a_1_, a_2_ and a_3_). However, on the soaked and then steamed nuts (Fig. 4c_1_, c_2_ and c_3_) a clear space appears between the kernel and the shell. This gap is maintained during the subsequent drying process (Fig. 4d_1_, d_2_ and d_3_).

#### Changes in whole nut, kernel and shell volumes following pre-treatment operations

Microtomography can also be used to quantify the volumes of the whole nut, the kernel and the shell. The evolution of these volumes following pre-treatment operations, estimated with a 70% confidence interval, is shown in Fig. 3b. Unfortunately, the large experimental errors did not allow us to observe any significant differences in volumes following the soaking, steaming and drying operations, although the spaces between the shell and kernel are clearly visible on the cross-sections. Consequently, further work would be required to minimise these errors, which are probably due to the methodology (orientation of the nut, cross-section for quantification, etc.). Thus, although microtomography provides highly relevant information in qualitative terms, its quantitative use remains delicate due to the uncertainties generated.

### Discussion

Water content emerged as the main factor influencing whole kernel yield, shelling rate and the breaking strength of nut shells. Increasing the water content by immersion and/or steaming is correlated with a concomitant increase in the yield and production rate of whole kernels, while a decrease in the breaking strength of nut shells is observed.

During soaking, the shell absorbs a lot of water, whereas the kernel absorbs very little. This increase in the water content of the shell, which has a lignocellulosic structure similar to that of wood (Tkatchenko 1949; de Paiva et al. 2024), causes its fibres to swell. This expansion of the volume of the shell while the volume of the kernel remains unchanged leads to a partial detachment of the kernel from the internal surface of the shell (visible by microtomography), making it easier to separate the kernel from the shell during shelling.

The hydro-thermal conditions of the steaming process cause the shell to soften. This softening is explained by structural and chemical changes in hemicellulose, lignin and cellulose, which represent approximately 58%, 24% and 13% (dry basis without ash) of the cashew nut shell respectively (de Paiva et al. 2024), under the combined effect of the rise in temperature and water content (Candelier 2013; Pelaez-Samaniego et al. 2013; Hon and Shiraishi 2000). More specifically, in the range of the nut water content considered (7.7–20.0% db), lignin and hemicellulose have softening temperatures of around 72 to 128 °C and 54 to 142 °C respectively, while that of cellulose exceeds 200 °C (Hon and Shiraishi 2000). Thus, at a steaming temperature of 100 °C (which was used in our study) the hemicelluloses, which are less polymerised and more reactive, degrade first because of their thermal sensitivity, while lignin mainly undergoes structural changes. In contrast, cellulose, which is more thermally stable, is little affected by these treatment conditions (Candelier 2013; Pelaez-Samaniego et al. 2013; Hon and Shiraishi 2000). Consequently, these thermal alterations in the wet environment of the lignocellulosic structure of the shell lead to its breakdown during steaming, making it more brittle after drying. This modification facilitates shelling.

Steaming also leads to changes in the cashew kernel due to the starch it contains, which represents 20 to 25% of its mass (Tkatchenko 1949; Chen et al. 2022). Under the combined effect of high temperature (100 °C) and water content, the starch undergoes gelatinisation (Briffaz et al. 2012; Bahrani 2012), given that the gelatinisation temperature of cashew kernel starch is around 95 °C (Chen et al. 2022). Then, during the drying of the steamed nuts, which takes place at a lower temperature, the starch cools and undergoes a retrogradation mechanism. These irreversible structural and physico-chemical changes result in an amorphous structure that is more resistant to the mechanical stresses of shelling.

During the drying operation, the applied temperature does not exhibit a significant effect on the shelling performance within the studied range (30 to 60 °C). However, this artificial drying method (distinct from the current natural drying conducted at artisanal and semi-industrial scales) allows for surface dehydration of the shell while keeping the kernel hydrated. The shell, undergoing significant water loss, contracts, hardens, and becomes more fragile, while the kernel, remaining hydrated, maintains its resistance to breakage during shelling. Given that during steaming, the kernel partially detaches from the shell, these two mechanisms promote the obtention of whole kernels. Moreover, artificial drying is much faster than natural drying and reduces the total duration of pre-treatment operations by half.

To assess changes in physical properties, the volumes of nuts, kernels and shells were identified during pre-treatment using microtomography to measure changes in length, width and thickness. However, the results obtained were of the order of magnitude of the experimental errors, preventing significant quantification of dimensional changes and suggesting small dimensional changes. Despite this limitation, microtomography provided valuable structural information, particularly on the detachment of the kernel and the weakening of the shell.

Microtomographic imaging, which has never been used in previous studies on this subject, clearly revealed the structural changes in the shell and kernel during pre-treatment. It highlights how soaking, steaming, and drying facilitate kernel detachment and shell weakening, leading to improved shelling efficiency. Additionally, this study uncovers lignocellulosic transformations that enhance shell brittleness post-drying and starch modifications that improve kernel resilience to mechanical stress. Furthermore, artificial drying is introduced as a faster, controlled alternative to traditional drying methods, reducing processing time while preserving kernel integrity. These findings establish a scientific foundation for optimizing industrial cashew processing, improving both efficiency and product quality.

## Conclusion

This study has enabled us to gain a better understanding of the mechanisms involved in the immersion, steaming and drying pre-treatments that facilitate the shelling operation. It has shown that the shell hydration operation is decisive, but that the water immersion operation can be dispensed with and that the steaming operation is sufficient to saturate the shell’s fibrous structure with water. The hydration of the shell is therefore necessary to encourage the partial transfer of this water from the shell to the kernel in contact with it and thus soften the kernel. Steaming and drying partially detach the kernel from the shell and then, drying the shell quickly and superficially makes it brittle. The steaming and drying stages are therefore crucial for obtaining a reduced force during shelling, preserving the integrity of the kernels and guaranteeing a high shelling rate. The duration of steaming at 100 °C is also an important parameter for high-speed production of whole kernels. Finally, artificial drying considerably reduces the overall duration of the process.

In order to confirm the water and heat transfer mechanisms during these pre-treatments and their impact on weakening the shell and obtaining whole kernels, it would be relevant to model the process. Simulation would also make it easier to find the optimum processing conditions to increase the yield of whole kernels.

## Data Availability

The data and materials used in this study are available from the corresponding author upon reasonable request.

## References

[CR1] Azam-Ali SH, Judge EC (2001) Small-scale cashew nut processing. FA0. http://www.fao.org/3/a-ac306e.pdf. Accessed 28 May 2024

[CR2] Bahrani B, S. A (2012) Modification des propriétés physico-chimiques de l’amidon par procédés hydrothermiques: Contribution à l’étude des transferts couplés chaleur-masse. [Université de La Rochelle]. https://theses.hal.science/tel-00823904

[CR3] Balasubramanian D (2001) Physical properties of Raw cashew nut. J Agric Eng Res 78:291–297. 10.1006/jaer.2000.0603

[CR5] Balasubramanian D (2006) Improving whole kernel recovery in cashew nut processing specific to Nigeria nuts. *Agricultural mechanization in Asia, Africa and Latin America*. 37:58–64

[CR4] Balasubramanian D (2015) Failure force and energy of in-shell cashew nuts under uni-axial compression loading influenced by moisture content. J Bioresource Eng Technol 02:08–16

[CR6] Briffaz A, Mestres C, Escoute J, Lartaud M, Dornier M (2012) Starch gelatinization distribution and peripheral cell disruption in cooking rice grains monitored by microscopy. J Cereal Sci 56:699–705. 10.1016/j.jcs.2012.09.001

[CR7] BS ISO 11093-3:1994 (1995) Paper and board. Testing of cores. Part 3 determination of moisture content using the oven drying method. British Standards Institution

[CR8] Candelier K (2013) *Caractérisation des transformations physico-chimiques intervenant lors de la thermodégradation du bois. Influence de l’intensité de traitement, de l’essence et de l’atmosphère* [Université de Lorraine]. https://hal.univ-lorraine.fr/tel-01750346

[CR9] Chen N, Wang Q, Wang M-X, Li N, Briones AV, Cassani L, Prieto MA, Carandang MB, Liu C, Gu C-M, Sun J-Y (2022) Characterization of the physicochemical, thermal and rheological properties of cashew kernel starch. Food Chem. 10.1016/j.fochx.2022.100432 PMC9532777 36211743

[CR10] de Paiva EM, Mattos ALA, da Silva JCG, Mumbach GD, Arias S, Pacheco JGA, Di Domenico M, Alves JLF, de Brito ES (2024) Pyrolysis of cashew nutshell residues for bioenergy and renewable chemicals: kinetics, thermodynamics, and volatile products. J Anal Appl Pyrol. 10.1016/j.jaap.2023.106303

[CR11] Dhanushkodi S, Wilson VH, Sudhakar K (2016) Energy analysis of cashew nut processing agro industries: A case study. Bulgarian J Agricultural Sci 22:635–642

[CR12] Hon DNS, Shiraishi N (2000) Wood and cellulosic chemistry, revised, and expanded, 2nd edn. CRC. 10.1201/9781482269741

[CR13] INC (2020) Nuts & dried fruits—Statistical yearbook 2019/2020. International Nuts and Dried Fruits Concil. https://inc.nutfruit.org/wp-content/uploads/2021/09/1594640174_INC_Statistical_Yearbook_2019-2020.pdf

[CR14] Kilanko O, Ojolo SJ, Leramo RO, Ilori TA, Sunday O, Oyedepo PO, Babalola, Ojo SI, Fayomi PN, Onwordi Edidiong Ufot, et Akaninyene Ekwere. (2020) « dataset on physical properties of Raw and roasted cashew nuts ». Data Brief 33:106514. 10.1016/j.dib.2020.106514 PMC7683228 33251309

[CR15] Kolliesuah NP, Saysay JL, Zinnah MM, Freeman AT, Chinenye D (2020) Trend analysis of production, consumption and export of cashew crop in West Africa. Afr Crop Sci J 28:187–202. 10.4314/acsj.v28i1.14S

[CR16] Kosoko SB, Sanni LO, Adebowale AA, Daramola AO, Oyelakin MO (2009) Effect of period of steaming and drying temperature on chemical properties of cashew nut. Afr J Food Sci 03:156–164

[CR17] Lautié E, Dornier M, de Souza Filho M, Reynes M (2001) Les produits de L’anacardier: caractéristiques, voies de valorisation et marchés. Fruits 56:235–248. 10.1051/fruits:2001126

[CR18] Mah E, Schulz JA, Kaden VN, Lawless AL, Rotor J, Mantilla LB, Liska DJ (2017) Cashew consumption reduces total and LDL cholesterol: A randomized, crossover, controlled-feeding trial. Am J Clin Nutr 105:1070–1078. 10.3945/ajcn.116.150037 28356271

[CR19] Mandal S (2019) Biochemical profile of cashew nut. Int J Experimental Res Rev 20:48–50. 10.52756/ijerr.2019.v20.005

[CR20] Mohod A, Jain S, Powar AG (2010) Pollution sources and standards of cashew nut processing. Am J Environ Sci 06:324–328. 10.3844/ajessp.2010.324.328

[CR21] Muniz CR, Freire FCO, Soares AA, Cooke PH, Guedes MIF (2013) The ultrastructure of shelled and unshelled cashew nuts. Micron. 10.1016/j.micron.2013.08.006 24045033

[CR22] Ogunsina BS (2013) Crackability and chemical composition of pre-treated cashew nuts using a hand-operated knife cutter. Agricultural Eng International: CIGR J 15:275–283

[CR23] Ogunsina BS, Bamgboye AI (2014) Pre-shelling parameters and conditions that influence the whole kernel out-turn of steam-boiled cashew nuts. J Saudi Soc Agricultural Sci 13:29–34. 10.1016/j.jssas.2012.12.005

[CR24] Pelaez-Samaniego MR, Yadama V, Lowell E, Espinoza-Herrera R (2013) A review of wood thermal pretreatments to improve wood composite properties. Wood Sci Technol 47:1285–1319. 10.1007/s00226-013-0574-3

[CR25] Ricau P (2019) *The West african cashew Sector in 2018: General trends and country profiles*. Nitidae. https://www.nitidae.org/files/41dc7432/wa_cashew_sector_review_2019_nitidae.pdf. Accessed 13 November 2023

[CR26] Tkatchenko B (1949) L’anacardier. Fruits d’Outre-Mer 4:241–248

[CR27] Tran SD, Tran TTT, Tong MC, Uchiyama N (2020) Optimization of steaming, drying, and tempering pretreatments for improving shelling of extra-large cashew nuts. *Proceedings of the Institution of Mechanical Engineers, Part C: Journal of Mechanical Engineering Science*. 10.1177/0954406219876966

